# Experiences in a Metropolitan Asylums Board Hospital

**Published:** 1913-08-30

**Authors:** 


### HOSPITALS FROM THE PATIENTS' POINT OF VIEW.

(Contributions to this Section are Invited.)

### Experiences in a Metropolitan Asylums Board Hospital.

Twelve months ago I chanced to be in London in connection with a lawsuit in my capacity as a solicitor, and I was staying at a West-End hotel. Although adults are usually thought to be comparatively immune, I was unfortunate enough in some mysterious manner to contract scarlet fever. When the doctor who was called in announced his diagnosis to the hotel authorities there was naturally no small consternation, the place being full of visitors of all ages at the time, and before I could think out any alternative plan (being, indeed, half delirious), I found my bedroom invaded by two stalwart stretcher-bearers in the uniform of the Metropolitan Asylums Board, who were ushered in by the medical gentleman aforesaid, the rear being brought up by a very deaf nurse, who informed me that she was "taking ambulance duty" and had instructions to remove me at once to the —— Hospital. Resistance being clearly out of the question, I submitted to the nurse depriving me of my pyjamas and wrapping me up in the hospital blankets, whereupon I was hurried down a side staircase by the stretcher-bearers, the doctor assuring a few scared-looking visitors whom he encountered that I was "a case of appendicitis, being removed for operation." The ambulance was a large motor-driven affair, and the nurse sat by my side, with restoratives to hand in case of need, constantly inquiring if I was warm and comfortable; and thus, without noise, rattle, or jar, I was swiftly conveyed to a public fever hospital.

The ambulance drew up at the door of the receiving room, and in two minutes I was on a bed in a warm room having my temperature taken. Then the receiving doctor made a brief examination, confirmed the previous diagnosis, and after chatting for a few minutes asked me if I would like to have a ward to myself. Now these hospitals run by the Metropolitan Asylums Board are paid for out of the rates, and are, of course, instituted for the public safety and for the purpose of trying to stamp out infectious diseases in the populated areas. No payment, therefore, is expected, and it is unusual for anyone other than working-class people to be admitted, as people of means generally prefer to pay for the extra comforts and attention that they would expect from a private institution such as the London Fever Hospital. This explanation is necessary in view of the fact that a private ward was offered to me, this privilege being presumably a matter of courtesy. The proper use of these "private" wards, which are small side wards in the same block as, but separated from, the large wards, is for patients who have two complaints; for example, scarlet fever and ringworm, or measles and whooping-cough. It is needless to add that I gladly accepted this kind offer of the doctor's and that it made all the difference in the world to my comfort, as my friends and relations were able, though warned that they did so at their own risk, to visit me, and I was able to enjoy the privacy and freedom from the noise that were greatly coveted by some of the patients of the large wards, where at times delirious cases kept the rest awake.

### Diets and Patients' Criticisms.

My illness ran an easy and normal course. I lived for the first few days on milk and soda-and-milk, and as soon as the fever abated and the high temperature (mine was 105 on admission) dropped this was supplemented by milk puddings and bread and butter, and after a week or ten days I was put on full diet, which was as follows:—

Breakfast.—Bread and butter (excellent bread and dairy butter), a boiled egg (I am a connoisseur in eggs, and never once did I have one that was anything but perfectly fresh), and tea. Supply quite unlimited. About eleven a glass of milk. Dinner at 12.30: Roast or boiled chicken, with two vegetables; and milk or custard pudding, stewed fruit. Tea at 4.—Tea, bread and butter, and jam.

Supper at 7.—Milk pudding or calf's-foot jelly.

Whereas I, accustomed to good food, was perfectly satisfied with the hospital fare, and, in fact, thought it excellent, the occupants of the big wards, mainly from the slums, kept up a continual grumble, and were always complaining to me when I got up later and moved among them of the bad quality of the food and its monotony. Upon comparing notes, however, I found that they got exactly the same as myself. Personally, I thought at the time, and I think still, that it speaks volumes for the wonderful management of this huge institution that I was never once in six weeks offered any food which was not of the highest quality.

The month I spent in bed passed very quickly. At 6.30 a.m. beds were made—too early I think was the general opinion—and by eight the day nurses and the ward sister had taken the place of the night nurse. At about ten the doctor assigned to the ward made his rounds, and a visit was paid also nearly every day by the medical superintendent. This official is at the head of the whole staff and must be a very busy man. He visited every ward as a rule—measles, scarlet fever, enteric, diphtheria, etc. He wore no white coat like the ward doctor, and it seemed to me that there was some danger of his carrying infection from one class of cases to another, but I never heard of any specific instance of this occurring. The matron, too, paid frequent visits during the daytime, and was occasionally accompanied by a member of the visiting committee, who looked very much like a fish out of water.

If the case was going on well the daily visits of the doctor were purely perfunctory, except that once a week a thorough examination was made and the state of the patient entered up on his bed board. On this board is hung a printed form giving the history of the case, name, address, address of parent or guardian, date of admission, and so on, and at the foot a most gruesome anticipation—"Date of death!" I am glad to say that the mortality from scarlet fever seems to be low. I only heard of one death while I was a patient in the "scarlet" wards, whereas there were several from enteric during the same period.

The afternoon is given up to writing letters (all letters sent out are carefully sterilised) and reading, and by 8 p.m., when the night nurse comes on duty, patients are supposed to be in bed and asleep. The ward doctor pays a second visit at about 10 p.m., but does not enter the ward unless any case is in a serious condition. A night sister makes two or three rounds of every ward during the night to see that all is well, and that the night nurse has not fallen asleep at her post, and the wards are connected by telephone with the office. If any patient is seriously ill an extra night nurse is provided.

### Two Types of Nurse.

Although the month was August, I suffered a good deal from cold and shivering, and my night nurse was untiring in fetching hot blankets and water-bottles, lighting my fire, and feeding me on Benger's Food at intervals. In fact, no words of mine can praise the nurses sufficiently as a body, though there were certainly exceptions, and one of my day nurses was a very rough woman, with a clumsy manner and a harsh, unpleasant voice, quite unsuited to nursing at all, and especially unfitted for a ward where the great majority were children. These went about in terror of her, and, she was notorious all over the hospital for her ways.

It was just under a month from the date of my entrance into hospital that I was ordered up, and, strange to say, I was most unwilling to comply. I felt weak and peaceful and should have preferred to stay in bed indefinitely. Usually, I believe, the case is quite the reverse and patients clamour to be allowed up, with the result that the doctor sometimes gives his permission rather too early for safety, and the result is not infrequently a relapse, nephritis, and perhaps Bright's disease. Several instances of this came under my notice, and it seems to me that this is a matter in which the authorities should show more caution.

I found, on mingling with the other patients, that bed was very irksome to them and that they all eagerly looked forward to the earliest possible date at which they might be allowed to get up. This was probably explained by the fact that they cared little for reading, although a library of miscellaneous books—Dickens, Marryat, "Robinson Crusoe," and "Gulliver's Travels" caught my eye—is provided for each ward.

Then, too, for those who are up there are draughts, dominoes, chess, cards, and a bagatelle board; and outside each ward a concreted court affords a space for cricket, tennis, and so on. A covered-in verandah and balcony run round the entire length of the ward, and here the convalescents could exercise even in wet weather.

### The Rules and the Doctor.

After patients have been up about a week or so they are usually sent off to one of the country establishments used by the Metropolitan Asylums Board as convalescent homes, but I had heard such adverse rumours about the comfort of these places that I got permission to stay in my own little ward until I was able to be discharged cured and non-infectious. The wards at the convalescent homes, I was told, were crowded, fifty beds to a ward instead of twenty, the food coarse, and the management bad. But this is mere hearsay, and therefore unreliable. The large ward was a very bright, clean place, airy, devoid of square corners, with windows on both sides, and French windows opening on to the verandah at the end. There were always vases of flowers in profusion. Visitors were allowed by special permit to enter the gates and proceed up the drive a few yards to exchange a few words at a distance with any of their relatives who were well enough to be outside on the verandah. But the orders to the gate porter were very stringent on this subject, and I heard many complaints about friends being refused admission after a long journey. It is clear, however, that these rules are really very necessary, for my brother, a medical man of many years' standing, visited me in my private ward and caught the disease himself, and was removed to another Metropolitan Asylums Board institution, where, curiously enough, he had previously been on the medical staff.

After patients have been up a few days the medical superintendent or his assistant examines them, and they are then drafted to the convalescent homes as vacancies occur. They are transferred by a special hospital motor-bus, having previously had a bath and a change of clothing. Upon their arrival in the country these clothes are taken away and a third set provided. The patients then remain ten days or a fortnight until the peeling process has entirely ceased, when they are taken to a changing room, bathed, and put into their own clothes and discharged. Notwithstanding all these elaborate precautions "return cases" constantly occur, and one boy in the big ward near me had had an attack of scarlet fever, spent a month in hospital, and a fortnight in a convalescent home, and two or three days after his discharge was readmitted suffering from a second attack. Obviously in his case his house had not been properly disinfected on his first removal.

Upon my release from hospital I merely had a bath, put on my own clothes in lieu of the hospital outfit, had my books, papers, watch and chain, etc., disinfected, and my money restored to me (no money is allowed to be handled by patients), and walked out. It is often stated that a scarlet-fever patient remains infectious until desquamation has entirely ceased, but "peeling" in my case continued for three weeks after I left hospital, or nine weeks from the time I contracted the disease. Although I was staying during this latter period at a dairy farm where there were children, no case of scarlet fever was reported, either in the farm or the village.

